# Unpredictable In Vitro Killing Activity of Amphotericin B against Four *Candida auris* Clades

**DOI:** 10.3390/pathogens10080990

**Published:** 2021-08-06

**Authors:** Zoltán Papp, Andrew M. Borman, Lajos Forgács, Renátó Kovács, Zoltán Tóth, Chiu Chun-Ju, Gábor Kardos, Béla Juhász, Judit Szilvássy, László Majoros

**Affiliations:** 1 Department of Otorhinolaryngology and Head and Neck Surgery, University of Debrecen, 4032 Debrecen, Hungary; myamy2006@gmail.com (Z.P.); szj@med.unideb.hu (J.S.); 2 UK National Mycology Reference Laboratory, Public Health England, Science Quarter, Southmead Hospital, Bristol BS10 5NB, UK; Andy.Borman@nbt.nhs.uk; 3 Medical Research Council Centre for Medical Mycology (MRC CMM), University of Exeter, Exeter EX4 4QD, UK; 4 Department of Medical Microbiology, Faculty of Medicine, University of Debrecen, 4032 Debrecen, Hungary; forgacs.lajos.89@gmail.com (L.F.); kovacs.renato@med.unideb.hu (R.K.); toth.zoltan@med.unideb.hu (Z.T.); junchiou02@gmail.com (C.C.-J.); kg@med.unideb.hu (G.K.); 5 Doctoral School of Pharmaceutical Sciences, University of Debrecen, 4032 Debrecen, Hungary; 6 Faculty of Pharmacy, University of Debrecen, 4032 Debrecen, Hungary; 7 Department of Metagenomics, University of Debrecen, 4032 Debrecen, Hungary; 8 Department of Pharmacology and Pharmacotherapy, Faculty of Medicine, University of Debrecen, 4032 Debrecen, Hungary; juhasz.bela@med.unideb.hu

**Keywords:** *Candida auris*, amphotericin B, killing rate, time–kill, in vitro

## Abstract

*Candida auris* is an emerging multiresistant yeast against which amphotericin B (AMB) is still the first therapeutic choice in certain clinical situations (i.e., meningitis, endophthalmitis, and urinary tract infections). As data about the in vitro killing activity of AMB against *C. auris* clades are lacking, we determined MICs, minimum fungicidal concentrations (MFCs), and killing activity of AMB against 22 isolates representing the 4 major *C. auris* clades (South Asian n = 6; East Asian n = 4; South African n = 6, and South American n = 6). MIC values were ≤1 mg/L regardless of clades; MFC ranges were, 1–4 mg/L, 2–4 mg/L, 2 mg/L, and 2–8 mg/L for South Asian, East Asian, South African, and South American clades, respectively. AMB showed concentration-, clade-, and isolate-dependent killing activity. AMB was fungicidal at 1 mg/L against two of six, two of four, three of six, and one of six isolates from the South Asian, East Asian, South African, and South American clades, respectively. Widefield fluorescence microscopy showed cell number decreases at 1 mg/L AMB in cases of the South Asian, East Asian, and South African clades. These data draw attention to the weak killing activity of AMB against *C. auris* regardless of clades, even when MICs are low (≤1 mg/L). Thus, AMB efficacy is unpredictable in treatment of invasive *C. auris* infections.

## 1. Introduction

*Candida auris* is an opportunistic yeast that has emerged worldwide and causes superficial as well as life threatening infections among critically ill patients [1,2,3,4]. Currently, five phylogenetically distinct clades (South Asian, East Asian, South African, South American, and Iranian) have been identified by whole-genome sequencing, with significant genetic differences between clades [4,5]. Previous studies have shown that *C. auris* clades differ from each other in their virulence in *Galleria mellonella* and neutropenic murine models [6,7,8]. *C. auris* is able to produce large aggregates both in vitro and in vivo, a phenomenon which may be associated with its excellent ability to survive in different environmental conditions including the presence of antifungal agents [6,7,8,9]. High MICs to fluconazole, and to a lesser extent to new triazoles (i.e., posaconazole and isavuconazole), are common [10,11,12,13]. Currently, echinocandins (anidulafungin, caspofungin, and micafungin) are the recommended drugs for the treatment of invasive *C. auris* infection in individuals aged two months and older [14]. However, in patients who are unresponsive to echinocandin therapy or who have persistent candidemia, as well as in children younger than the age of two months, switching to liposomal or traditional amphotericin B (AMB) is recommended as initial therapy. Moreover, echinocandins penetrate poorly into the central nervous system, the eye, and the urinary tract; thus, in cases of meningitis, endophthalmitis, and urinary tract infections, AMB is still the first therapeutic choice [14,15,16,17].

Data on the in vitro killing activity of AMB against different *C. auris* clades are scant; thus, we have determined the killing activity of AMB against *C. auris* isolates belonging to the four prevalent clades.

## 2. Results

### 2.1. MIC Determination by Broth Microdilution and Etest Methods

Regardless of methods and clades, MIC values were never higher than the tentative susceptibility breakpoint (1 mg/L) suggested by the Centers for Disease Control and Prevention (CDC) (Table 1) [14]. Using the broth microdilution method (BMD) [18,19], the lowest (0.12–0.25 mg/L) and highest (0.5–1 mg/L) MIC value ranges were found for the East Asian and South Asian clades, respectively. MIC values of the 22 isolates with Etest after 24 and 48 h showed 77% (17/22) and 91% (20/22) agreement within ±one dilution compared to the BMD MICs after 24 h.

### 2.2. Minimum Fungicidal Concentrations (MFCs)

MICs obtained with higher starting inocula (~10^4^ CFU/mL) increased by 2–8-fold compared to the MICs obtained with standard BMD (~10^3^ CFU/mL), with the exception of isolate I-156 (South American clade) (Table 1). MFCs were uniformly 2 mg/L with the South African clade for all six isolates. MFC ranges were 1–4 mg/L, 2–4 mg/L, and 2–8 mg/L for South Asian, East Asian, and South American clades, respectively (Table 2). The MFC per MIC ratios were 2–4, 8–32, 4–8, and 4–16 for South Asian, East Asian, South African, and South American clades, respectively.

### 2.3. Time Kill Results

**Figure 1:**
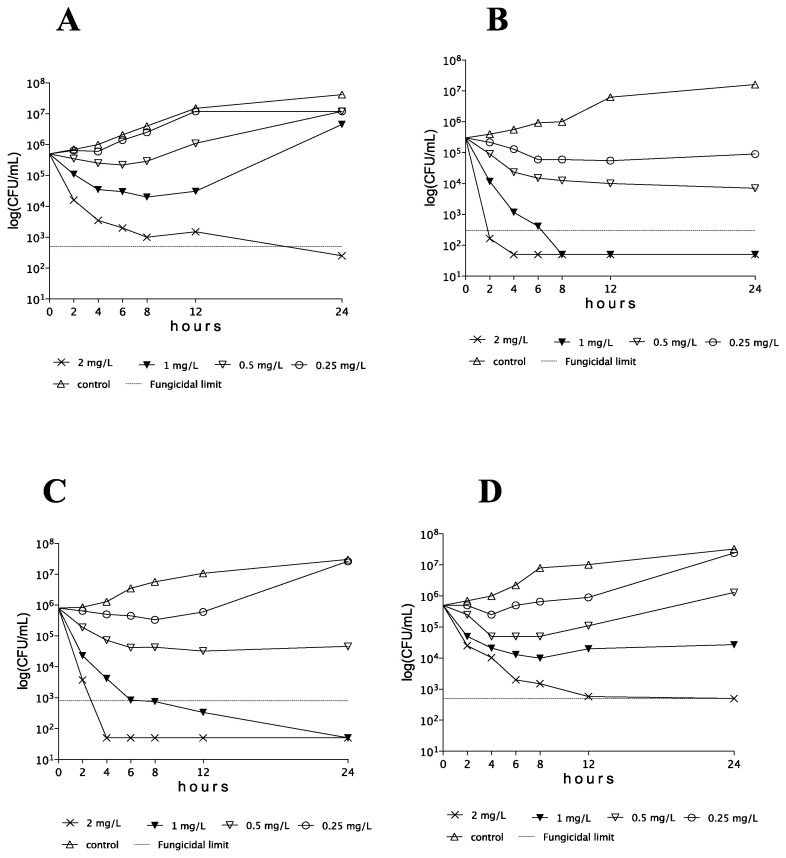
Time–kill plots of amphotericin B against *Candida auris* isolates 174 (**A**), type strain (NCPF 13029 = CBS 10913) (**B**), 182 (**C**), and I-172 (**D**), belonging to the South Asian, East Asian, South African, and South American clades, respectively.

AMB showed concentration-dependent killing activity against *C. auris* isolates at ≥0.5 × MIC. However, CFU decreases were frequently transient, with regrowth even at 2 × MICs after 12 h. Representative time–kill plots with each clade are shown in Figure 1.

#### 2.3.1. South Asian Clade

**Figure 2:**
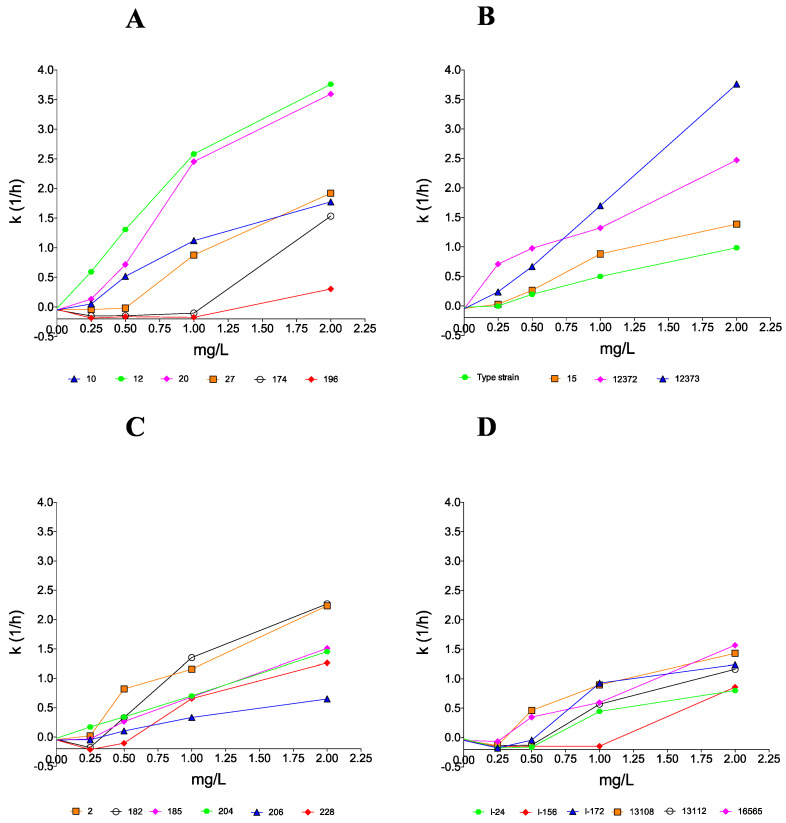
Killing rate values of amphotericin B against 22 clinical isolates belonging to the South Asian (**A**), East Asian (**B**), South African (**C**), and South American (**D**) *Candida auris* clades. Positive and negative *k* values indicate the decrease and increase, respectively, in viable cell numbers. Error bars are omitted for better visualization of the graphics.

AMB proved to be fungicidal at 1 and 2 mg/L against 2 of 6 and 5 of 6 clinical isolates, respectively. In the cases of isolates 174 and 196 at MICs (1 mg/L), CFU decreases were detected only in the first 8–12 h (Table 2), followed by prominent regrowth and negative *k* values in the case of both isolates (Figure 2). Although AMB was fungicidal at 2 mg/L (T99.9 = 1.96 h) in the case of isolate 174 (Table 3), only a weak fungistatic effect (−1.22-log CFU/mL decrease) was found with isolate 196 (MFC was 4 mg/L) (Table 2).

#### 2.3.2. East Asian Clade

AMB at ≥0.5 mg/L generated CFU decreases without regrowth with all isolates of this clade. The type strain was killed at 1 mg/L (4 × MIC) after 1.77 h. In spite of the higher MFC (4 mg/L) against isolate 12372, AMB produced fungicidal effects at ≥0.25 mg/L (≥2 × MIC) (T99.9 ranges were 1.21–4.24 h, Table 3). Isolates 15 and 12373 were killed at their MFCs (2 mg/L) (T99.9 were 0.79 and 3.05 h, respectively).

#### 2.3.3. South African Clade

AMB at 1 and 2 mg/L produced a fungicidal effect against 3 of 6 and 5 of 6 clinical isolates, respectively. Isolate 182 showed regrowth at 2 × MIC (0.5 mg/L) but was killed at ≥1 mg/L (≥4 × MIC) after 1.32–2.22 h (Table 3). The maximum CFU decreases at 1 mg/L were not higher than 2.41-log for isolates 185, 206, and 228 (Table 3). Although the MFC for isolate 228 was 2 mg/L, at 2 mg/L (4 × MIC) CFUs decreased only by 2.50-log.

#### 2.3.4. South American Clade

All isolates at 1 × MICs and frequently at 2 × MICs showed regrowth after 24 h. At 1 mg/L, only isolate 13108 was killed (T99.9 was 3.35 h) and five of six isolates did not reach even the 99% growth reduction compared to the starting inoculum (Table 3). Although the three environmental isolates were killed at their MFCs (2 mg/L) (T99.9 values were 1.91–2.58 h), two bloodstream isolates from Israel (I-24 and I-156) did not reach the 99% growth reduction. The mean *k* value in the case of isolate I-156 was positive only at 2 mg/L (k = 0.859 1/h); however, after 12 h, regrowth was observed (Figure 2).

### 2.4. Widefield Fluorescence Microscopy

**Figure 3:**
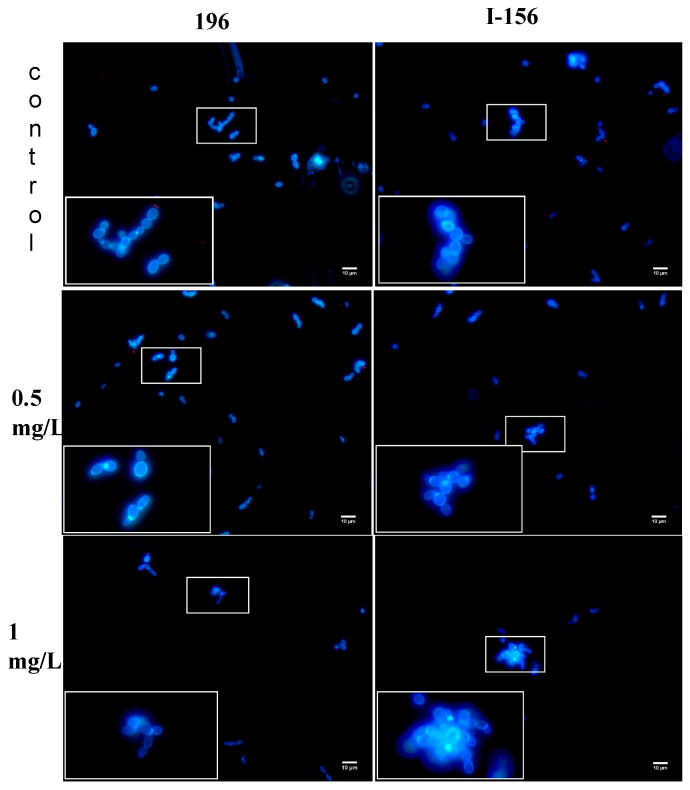
*Candida auris* cells (isolates 196, South Asian clade and I-156, South American clade) treated with 0.5 and 1 mg/L amphotericin B and examined with widefield fluorescence microscopy. Drug-free controls were included. Micrographs were taken after 24 h. Blue cells indicate viable cells. In the case of isolate 196 (**left**), cell numbers decreased at 1 mg/L. For isolate I-156 (**right**), large aggregates with up to 50 cells were seen at 1 mg/L. Dead cells were never detected. Bar: 10 µm.

In controls, single and budding cells or short chains were seen with isolates 196, 15, and 228. In the case of isolate I-156, the appearance of the untreated isolate was similar, but small aggregates (up to 10–15 cells) were frequently observed. AMB-treated *C. auris* cells showed similar morphology and cell number decreases with isolates 196, 15, and 228 at 0.25–1 mg/L; however, in the case of isolate I-156 large (20 µm in diameter), aggregates with up to 50 cells were seen at 1 mg/L (Figure 3). Uptake of PI, indicating the presence of dead cells, was never observed.

## 3. Discussion

Regardless of clades and isolates, MICs with the standard BMD and Etest (both after 24 and 48 h) methods were not higher than the tentative breakpoint suggested by the CDC for *C. auris* [14]. AMB proved to be fungicidal in MFC tests at 1–8 mg/L after 24 h against the four prevalent *C. auris* clades; the MFC values were 2–32-fold higher than the corresponding MICs obtained with the BMD methodology, indicating a fungistatic action at concentrations around the MIC. Importantly, with the exception of isolate 20 (South Asian clade), these MFCs were higher than the clinically attainable AMB concentration (1 mg/L) in the serum [20]. In our killing studies, AMB showed concentration-, clade-, and isolate-dependent killing activity against *C. auris*. MFC results with the exceptions of isolates 12372 (East Asian clade), 228 (South African clade) and I-172 (South American clade) showed good correlation with the time–kill results (Table 2).

AMB at the clinically attainable 1 mg/L proved to be fungicidal against only two of six, two of four, three of six, and one of six of the isolates from the South Asian, East Asian, South African, and South American clades, respectively. Moreover, two of six, and three of six isolates from the South Asian and South American clades, respectively, showed regrowth at 1 mg/L. Widefield fluorescence microscopy showed cell number decreases with isolates 196, 15, and 228, while, in the case of isolate I-156, prominent aggregates were found at 1 mg/L (Figure 3), but dead cells were never detected. Taken together, these data indicate that, at clinically attainable concentrations, AMB is not reliably fungicidal against any of the *C. auris* clades studied.

Data about the in vitro killing activity of AMB against *C. auris* are scant; nine bloodstream isolates from Colombia were studied earlier [21]. In that study, AMB was fungicidal at 2–4 mg/L (1–4 × MIC) in MFC tests and showed concentration-dependent but isolate-independent killing activity at >2 mg/L in time–kill tests. The T99.9 value ranges were significantly longer (from 3.3 to 11.7 h, the corresponding *k* values were from 0.256 to 0.913 1/h), than the T99.9 values of our four of six South American isolates at 2 mg/L. However, that study only reported an averaged *k* value calculated using all drug concentrations taken together; thus, a single *k* value represented the average killing kinetics found at 0.12–8 mg/L for each strain [21]. It is noteworthy that, in the cases of our remaining two isolates (I-24 and I-156), the fungicidal endpoints were not archived at 2 mg/L (Table 2). The lack of prior studies of AMB killing activity against isolates from the South Asian, East Asian, and South African clades precluded comparison with our results.

The strength of our study is that the four main *C. auris* clades were compared using several clinical isolates of each clade. These findings need confirmation using in vivo models. Other authors found that 5 mg/kg of AMB against nine clinical isolates of *C. auris* belonging to the four clades produced mainly fungistatic activity (in the case of eight out of nine isolates, while one isolate showed growth) in a neutropenic murine model. Furthermore, a 1-log decrease was achieved only in three out of nine isolates [22]. In mice infected with isolates with high AMB MICs (2–4 mg/L), the fungal kidney tissue burden increased despite AMB treatment [22,23].

In this study, AMB was fungicidal against 16.7–50% of isolates from the four geographic clades at clinically attainable concentrations (≤1 mg/L). The background of the weak killing activity of AMB against our AMB non-resistant isolates is unknown; mutations in ergosterol biosynthesis genes have been reported earlier in such isolates, but not all AMB-resistant isolates exhibited such mutations [10]. In another study, mutations were not found in the ergosterol biosynthesis genes in AMB-resistant *C. auris* isolates from Colombia. Four alternative AMB resistance mechanisms were described, including mutations in genes encoding a transcription factor similar to FLU8 in yeasts and a putative membrane transporter [11]. Another possible mechanism is that alterations in the cell wall components, especially β-1,3-glucan or chitin can physically decrease the penetration of AMB into cells [12,17]. The decreased penetration of AMB into *C. auris* cells was supported with our isolate I-156, which produced prominent aggregates in the presence of 1 mg/L of AMB (Figure 3). Although the clinical relevance remains unknown, it is notable that the two clades (South Asian and South American) that had a greater resistance to AMB also proved to be the most virulent in a neutropenic murine model in our previous studies [8].

In summary, our MFC and time kill results draw attention to the weak killing activity of AMB against *C. auris* isolates regardless of clade, even when the MIC is low (≤1 mg/L). These data suggest that the efficacy of AMB for the treatment of invasive *C. auris* infections, including meningitis, endophthalmitis, and urinary tract infections, cannot be reliably predicted using MIC results, which may explain the high mortality with AMB treatment [3,4]. Although a combination of echinocandins with AMB may improve survival [13], the discovery of new antifungal agents with traditional or new targets is essential to improve the survival rate of infections by multiresistant fungi, including *C. auris* [24,25,26].

## 4. Materials and Methods

### 4.1. Isolates

Isolates of the four prevalent clades used (South Asian n = 6, East Asian n = 4, South African n = 6, and South American n = 6) are listed in Table 1. *C. auris* isolates were identified by a combination of ribosomal DNA gene sequencing targeting the 28S rRNA and/or ITS1 regions, which was also used for clade delineation [6,7,8,9]. Two days before the experiments, isolates were subcultured using Sabouraud agar and screened on CHROMagar^™^ Candida (Becton Dickinson) to ensure purity of *Candida* isolates.

### 4.2. Minimum Inhibitory Concentration

Isolates were tested by the broth microdilution method (BMD) according to CLSI M27-Ed4 document in RPMI-1640 (Sigma, Budapest, Hungary), with starting inocula of ~10^3^ CFU/mL. AMB was purchased from Sigma (Budapest, Hungary), dissolved in 100% DMSO and diluted further in RPMI-1640; MICs were read visually after 24 h incubation at 35 °C using the total inhibition criterion [18,19].

MIC values were also determined using the Etest method (AB BIODISK, Solna, Sweden) according to the instructions of the manufacturer, and the results were read after 24 and 48 h. If MIC values fell between the two-fold dilutions used in BMD, the value was rounded up to the next two-fold level of the BMD for comparison [27]. As a *C. auris*-specific susceptibility breakpoint for AMB is currently not established, we used the tentative MIC breakpoint suggested by the CDC (isolates with a MIC of ≥2 were considered resistant) [14].

### 4.3. Minimum Fungicidal Concentration

In MFC tests, starting inocula were ~10^4^ CFU/mL. MICs were read after 24 h; then, the entire content of each well containing supra-MIC concentrations was plated onto a drug-free Sabouraud dextrose agar [28].

### 4.4. Time–Kill Studies

Activity of AMB was determined in RPMI-1640 at concentrations 0.25, 0.5, 1, and 2 mg/L, in a final volume of 10 mL. The starting inocula were 3–8 × 10^5^ CFU/mL. Aliquots of 100 µL were removed after 0, 2, 4, 6, 8, 12, and 24 h of incubation, ten-fold serial dilutions were prepared, and samples of dilutions (4 × 30 µL) were plated onto a single Sabouraud dextrose agar plate and incubated at 35 °C for 48 h. All experiments were performed twice [9,24]. In MFC and time–kill tests, fungicidal activity was defined as a 99.9% reduction in viable CFU/mL compared to the starting inoculum [9,24]. Killing kinetics were analyzed as described previously. Briefly, an exponential equation was fitted to the mean data at six time points: N_t_ = N_0_ × e^−kt^, where N_t_ was the number of viable yeasts at time t, N_0_ was the number of viable yeasts in the initial inoculum, *k* was the killing rate, and *t* was the incubation time. Negative and positive *k* values indicate growth and killing, respectively. The goodness of fit for each isolate was assessed by the r^2^ value (r^2^ > ±0.8). The mean times to achieve the 99% (T99 = 2/*k*) and 99.9% (T99.9 = 3/*k*) reduction in viable cell count compared to that of the starting inoculum were calculated from the *k* values for each isolate and concentration [9].

### 4.5. Widefield Fluorescence Microscopy

AMB-induced morphological and viability alterations were examined using one isolate from each clade (isolates 196, 15, 228, and I-156). Yeasts were incubated at 37 °C with different AMB concentrations (0, 0.25, 0.5, and 1 mg/L) for 24 h; then, 979 µL of the culture was stained with 1 µL of 20 mM Propidium-iodide (PI) (ThermoFisher, Waltham, MA, USA) and 20 µL 3 mg/mL Calcoflour-white (CFW). Fluorescently stained cells were incubated further at 37 °C for 30 min; then, 10 µL of medium was mounted on a slide and examined using a Zeiss Axioskop 2 mot microscope coupled with a Zeiss Axiocam HRc camera to assess cell morphology and viability. Image acquisition was performed using the Zeiss Axiovision 4.8.2 software. CFW indiscriminately binds to the chitin content of the fungal cell wall, while PI only penetrates nonviable cells; therefore, this double staining technique is suitable to distinguish between viable and dead fungal cells.

## Figures and Tables

**Table 1:** MIC values of amphotericin B obtained with broth microdilution (BMD) and Etest methods against *Candida auris* isolates and type strain. For the Etest method, MICs were read both after 24 and 48 h.

Clade	Isolate Number	Body Site	MIC (mg/L)			
			BMD (CFU/mL)		Etest	
			10^3^	10^4^	24 h	48 h
**South Asian**	10	Wound swab	0.5	2	0.5	0.5
	12	Unknown	0.5	1	0.12	0.25
	20 (NCPF 8985)	Wound	0.5	1	0.25	0.5
	27 (NCPF 89891)	Pleural fluid	0.5	1	0.25	0.5
	174	Nose swab	1	2	0.25	1
	196	Blood	1	2	0.5	1
**East Asian**	15 (NCPF 8984)	External ear	0.25	1	0.25	0.5
	12372 (CBS 12372)	Blood	0.12	1	0.25	1
	12373 (CBS 12373)	Blood	0.25	1	0.25	1
	Type strain (NCPF 13029 = CBS 10913)	External ear	0.25	1	0.12	0.25
**South African**	2 (NCPF 8977)	Cerebrospinal fluid	0.5	1	0.25	0.5
	182	Sputum	0.25	2	0.5	0.5
	185	Blood	0.5	1	0.25	0.5
	204	Tracheostomy	0.5	1	0.25	0.5
	206	Blood	0.5	2	0.25	0.5
	228	Screening swab	0.5	2	0.5	1
**South American**	13108 (CDC B-13108)	Hospital environment	0.25	1	0.25	0.5
	13112 (CDC B-13112)	Hospital environment	0.5	1	0.12	0.25
	16565 (CDC B-16565)	Hospital environment	0.25	1	0.25	0.5
	I-24	Blood	1	2	0.25	1
	I-156	Blood	1	1	0.25	1
	I-172	Blood	0.5	2	0.25	0.5

**Table 2:** Minimum fungicidal concentrations (MFC) and maximum changes in log CFU/mL compared to starting inoculum in time–kill studies at different amphotericin B (AMB) concentrations (mg/L) and isolates of the four *Candida auris* clades. Data in bold indicate a fungicidal effect (at least 3-log decreases in CFU). NK: no killing.

Clade	Isolate Number	MFC (mg/L)	Maximum Log Decreases in CFU in Time–Kill Experiments at the Indicated AMB Concentration (mg/L)			
			0.25	0.5	1	2
**South Asian**	10	2	−0.43	−1.04	−1.9	−3.9
	12	2	−2.08	−2.3	−3.78	−3.78
	20	1	−0.74	−2.86	−3.78	−3.78
	27	2	−0.32 *	−1.0	−1.6	−3.6
	174	2	NK	−0.30 *	−1.20 *	−3.30
	196	4	NK	−0.18 *	−0.32 *	−1.22
**East Asian**	15	2	−0.46 *	−0.79	−1.47	−3.90
	12372	4	−3.26	−3.26	−3.78	−3.78
	12373	2	−0.38 *	−1.22	−1.88	−3.78
	Type strain	2	−0.74 *	−1.60	−3.78	−3.78
**South African**	2	2	−0.40 *	−1.62	−3.78	−3.78
	182	2	−0.38 *	−1.39 *	−4.20	−4.20
	185	2	−0.32 *	−0.90	−2.23	−3.90
	204	2	−1.09	−2.38	−3.78	−3.78
	206	2	−0.30 *	−1.03	−2.41	−4.15
	228	2	NK	−0.46 *	−1.48	−2.50
**South American**	13108	2	−0.48 *	−1.78 *	−3.78	−3.78
	13112	2	−0.32 *	−0.96*	−1.78	−3.60
	16565	2	−0.88 *	−1.18 *	−1.82	−3.08
	I−24	8	NK	−0.79 *	−1.05 *	−1.85
	I−156	4	NK	−0.25 *	−0.63 *	−1.40 *
	I−172	8	−0.30 *	−1.00 *	−1.70 *	−3.00

* Regrowth.

**Table 3:** Time (hours) to reach 99% (T99) and 99.9% (T99.9) growth reduction from the starting inocula at different amphotericin B concentrations (mg/L).

Clade	Isolate Number	Time (Hours)							
		T99				T99.9			
		0.25 mg/L	0.5 mg/L	1 mg/L	2 mg/L	0.25 mg/L	0.5 mg/L	1 mg/L	2 mg/L
**South Asian**	10	* NA	NA	1.79	1.12	NA	NA	NA	1.69
	12	3.36	1.53	0.78	0.53	NA	NA	1.16	0.79
	20	NA	2.79	0.82	0.56	NA	NA	1.22	0.84
	27	NA	NA	NA	1.04	NA	NA	NA	1.56
	174	** NK	NK	NK	1.30	NK	NK	NK	1.96
	196	NK	NK	NK	NA	NK	NK	NK	NA
**East Asian**	15	NA	NA	NA	0.53	NA	NA	NA	0.79
	12372	2.82	2.05	1.51	0.81	4.24	3.08	2.27	1.21
	12373	NK	NA	NA	2.03	NK	NA	NA	3.05
	Type strain	NA	NA	1.18	0.53	NA	NA	1.77	0.79
**South African**	2	NA	NA	1.73	0.89	NA	NA	2.59	1.34
	182	NK	6.05	1.48	0.88	NK	NA	2.22	1.32
	185	NK	NA	2.93	1.33	NK	NA	NA	1.99
	204	NA	5.85	2.86	1.38	NA	NA	4.29	2.07
	206	NK	NA	6.00	3.08	NK	NA	NA	4.62
	228	NK	NK	NA	1.59	NK	NK	NA	NA
**South American**	13108	NK	NA	2.23	1.39	NK	NA	3.35	2.10
	13112	NK	NK	NA	1.72	NK	NK	NA	2.58
	16565	NK	NA	NA	1.28	NK	NA	NA	1.91
	I-24	NK	NK	NA	NA	NK	NK	NA	NA
	I-156	NK	NK	NK	NA	NK	NK	NK	NA
	I-172	NK	NK	NA	1.62	NK	NK	NA	2.42

* NA: not achieved. ** NK: no killing.

## Data Availability

Not applicable.
